# Metagenomic analysis of ecological niche overlap and community collapse in microbiome dynamics

**DOI:** 10.3389/fmicb.2023.1261137

**Published:** 2023-11-15

**Authors:** Hiroaki Fujita, Masayuki Ushio, Kenta Suzuki, Masato S. Abe, Masato Yamamichi, Yusuke Okazaki, Alberto Canarini, Ibuki Hayashi, Keitaro Fukushima, Shinji Fukuda, E. Toby Kiers, Hirokazu Toju

**Affiliations:** ^1^Center for Ecological Research, Kyoto University, Otsu, Shiga, Japan; ^2^Department of Ocean Science (OCES), The Hong Kong University of Science and Technology (HKUST), Kowloon, Hong Kong SAR, China; ^3^Integrated Bioresource Information Division, BioResource Research Center, RIKEN, Tsukuba, Ibaraki, Japan; ^4^Faculty of Culture and Information Science, Doshisha University, Kyotanabe, Kyoto, Japan; ^5^Center for Frontier Research, National Institute of Genetics, Mishima, Shizuoka, Japan; ^6^Department of International Health and Medical Anthropology, Institute of Tropical Medicine, Nagasaki University, Nagasaki, Japan; ^7^Institute for Chemical Research, Kyoto University, Uji, Kyoto, Japan; ^8^Faculty of Food and Agricultural Sciences, Fukushima University, Fukushima, Japan; ^9^Institute for Advanced Biosciences, Keio University, Tsuruoka, Yamagata, Japan; ^10^Gut Environmental Design Group, Kanagawa Institute of Industrial Science and Technology, Kawasaki, Kanagawa, Japan; ^11^Transborder Medical Research Center, University of Tsukuba, Tsukuba, Ibaraki, Japan; ^12^Laboratory for Regenerative Microbiology, Juntendo University Graduate School of Medicine, Tokyo, Japan; ^13^Department of Ecological Science, Vrije Universiteit Amsterdam, Amsterdam, Netherlands; ^14^Graduate School of Biostudies, Kyoto University, Sakyo, Kyoto, Japan

**Keywords:** community stability, competition, dysbiosis, ecological niche, metabolic interactions, shotgun metagenomics, microbial functions, time-series dynamics

## Abstract

Species utilizing the same resources often fail to coexist for extended periods of time. Such competitive exclusion mechanisms potentially underly microbiome dynamics, causing breakdowns of communities composed of species with similar genetic backgrounds of resource utilization. Although genes responsible for competitive exclusion among a small number of species have been investigated in pioneering studies, it remains a major challenge to integrate genomics and ecology for understanding stable coexistence in species-rich communities. Here, we examine whether community-scale analyses of functional gene redundancy can provide a useful platform for interpreting and predicting collapse of bacterial communities. Through 110-day time-series of experimental microbiome dynamics, we analyzed the metagenome-assembled genomes of co-occurring bacterial species. We then inferred ecological niche space based on the multivariate analysis of the genome compositions. The analysis allowed us to evaluate potential shifts in the level of niche overlap between species through time. We hypothesized that community-scale pressure of competitive exclusion could be evaluated by quantifying overlap of genetically determined resource-use profiles (metabolic pathway profiles) among coexisting species. We found that the degree of community compositional changes observed in the experimental microbiome was correlated with the magnitude of gene-repertoire overlaps among bacterial species, although the causation between the two variables deserves future extensive research. The metagenome-based analysis of genetic potential for competitive exclusion will help us forecast major events in microbiome dynamics such as sudden community collapse (i.e., dysbiosis).

## Introduction

Classic niche theory predicts that coexistence of species requires interspecific difference in resource use patterns (Volterra, 1928; Gause, 1934; Hardin, 1960; Zaret and Rand, 1971; Grime, 1973; Mayfield and Levine, 2010). Although some specific mechanisms can promote stable coexistence even with complete resource overlap (e.g., spatial structure of habitats and temporal variability in resource availability), similarity/dissimilarity in basic resource dependency among species is the basic factor determining the occurrence of competitive exclusion (Chesson, 2000, 2018; Letten et al., 2017). Therefore, evaluating the overlap of “fundamental niches,” which are defined by species’ fundamental resource requirements and resource-use capabilities (Hutchinson, 1957; Chase and Leibold, 2004), is an essential step for understanding and predicting community-level dynamics.

Insights into fundamental niches are encrypted in species’ genomes (Palomo et al., 2018; Smith et al., 2019; Régimbeau et al., 2022; Malard and Guisan, 2023): as species’ traits are encoded in their DNA, genomic information provides the ultimate basis for evaluating target species’ fundamental niches (Muller, 2019; Alneberg et al., 2020; Fahimipour and Gross, 2020). Thus, potential strength of competitive interactions within ecological guilds or communities could be evaluated based on the distribution of species’ gene repertoires within ecological niche space inferred with metagenomic data (Alneberg et al., 2020; Fahimipour and Gross, 2020; Herold et al., 2020; Régimbeau et al., 2022), also referred to as “metagenomic niche space.” Although overlap of niches does not always cause competitive exclusion (Chesson, 2000, 2018; Letten et al., 2017), higher levels of gene repertoire overlap within a community may impose greater impacts on population dynamics of constituent species.

In considering coexistence of microbial species, it is essential to examine whether such competition-driven population-level phenomena underly drastic ecological events observed at the community level. Microbial communities sometimes show sudden and substantial changes in species and/or taxonomic compositions (Ravel et al., 2013; Carding et al., 2015; Fujita et al., 2023b; Yajima et al., 2023). Human gut microbiomes, for example, have been reported to show drastic shifts from species-rich states to “imbalanced” states with low α-diversity and overrepresentation of pathogenic species (David et al., 2014; Lahti et al., 2014; Kho and Lal, 2018; Kriss et al., 2018) (e.g., *Clostridium difficile*). Elucidating the ecological mechanisms causing such drastic community-level events provide fundamental insights into microbiome dynamics (Costello et al., 2012; Huttenhower et al., 2012; Kriss et al., 2018). In this respect, an important challenge is to test the hypothesis that high levels of gene-repertoire overlap are observable prior to drastic community compositional changes. However, tests of this hypothesis have remained elusive due to the paucity of time-series observations of microbiomes with substantial compositional changes. Even if such microbiome time-series data are available, analyses of potential niche (gene repertoire) overlap require another line of information. Specifically, we need data of respective species’ genomes at multiple time points (Herold et al., 2020). Therefore, developing research systems that can overcome these constrains will deepen our understanding of microbiome ecological processes.

In this study, we examine the degree to which gene-repertoire overlap changes through dynamics of species-rich microbial communities. By targeting an experimental microbial system showing rapid and substantial changes in taxonomic compositions (Fujita et al., 2023b), we infer niche space depicting species’ gene repertoires. A previous study in this system using a metabolic modeling analysis suggested that interactions between species were keys to understand the drastic microbiome dynamics (Fujita et al., 2023a). Now, by compiling the shotgun metagenomic data collected at 13 time points across the 110-day time-series of the experiment, we reveal temporal shifts in the magnitude of gene repertoire overlap among microbial species. We then examine whether a high level of fundamental-niche overlap is observed prior to drastic changes in community structure. Overall, we explore how signs of drastic shifts in community structure are detected by inferring community-scale degree of fundamental niche overlap with the aid of genomic information. The knowledge will lead us to develop platforms for forecasting and preventing unfavorable shifts of microbiome compositions and those for recovering and designing functionally benign microbial ecosystems based on the evaluation of niche overlap levels.

## Materials and methods

### Time-series data of experimental microbiomes

We focused on the experimental microbiome showing drastic shifts in taxonomic compositions (Fujita et al., 2023b). In our previous study (Fujita et al., 2023b), a 110-day monitoring of microbiomes was performed with six experimental settings. To set up experimental microbiomes with high diversity of bacterial species/taxa, we used natural microbial communities derived from soil or pond-water ecosystems as source inocula, rather than “synthetic” communities with pre-defined diversity. In the experiment, microbiomes differing in the magnitude of community compositional shifts were constructed across the six treatments defined by the combinations of two inoculum source microbiomes and three types of media. One of the source microbiomes derived from the soil collected from the A layer (0–10 cm in depth) in the research forest of Center for Ecological Research, Kyoto University, Otsu, Japan (34.972°N; 135.958°E). The other source inoculum was prepared by collecting water from a pond (“Shoubuike”) near Center for Ecological Research (34.974°N, 135.966°E). Each of the source inocula was introduced into oatmeal (Medium-A), oatmeal-peptone (Medium-B), or peptone (Medium-C) broth media with eight replicates. Thus, in total, 48 experimental microcosms (two source microbiomes × three media × eight replicates) were constructed in a deep-well plate (1,000-μl-scale culture in each well). The plate was kept shaken at 1,000 rpm at 23°C. After 5-day pre-incubation, 200 μl out of the 1,000-μl culture medium was sampled from each well every 24 h for 110 days. In each sampling event, 200 μl of fresh medium was added to each well so that the total culture volume was kept constant. In total, 5,280 samples (48 communities/day × 110 days) were collected through the time-series experiment. After DNA extraction, the samples were subjected to the amplicon sequencing analysis of the 16S rRNA region (Fujita et al., 2023b).

**FIGURE 1:**
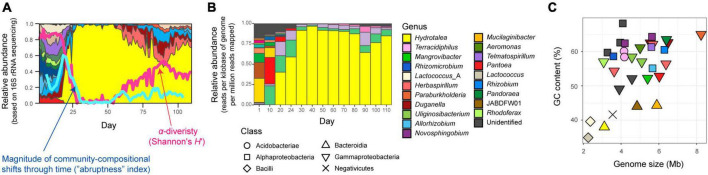
Community and ecosystem dynamics. **(A)** Time-series data of community structure. For the replicate microcosm that showed the most abrupt community compositional changes through the 110-day microbiome experiment (Fujita et al., 2023b; Supplementary Figure 1), family-level taxonomic compositions inferred with 16S rRNA sequencing are shown. The blue line represents the speed and magnitude of community compositional changes around each time point [“abruptness” index (Fujita et al., 2023b); see section “Materials and methods”]. The red line indicates α-diversity (Shannon’s *H’*) of microbial ASVs (Fujita et al., 2023b). Note that a value larger than 0.5 represents turnover of more than 50% of microbial ASV compositions. See Supplementary Figure 1 for color profiles of bacterial families. Reproduced from the data of a previous study (Fujita et al., 2023b). **(B)** Taxonomic compositions inferred with shotgun metagenomic sequencing. At each of the 13 time points through the time-series of the target microcosm, the relative abundance of each MAG was estimated based on the normalized read coverage value (reads per kilobase of genome per million reads mapped). **(C)** Genome size and GC nucleotide content of the MAGs detected in the target microcosm. See panel **(B)** for colors and symbols.

To quantify the speed and magnitude of community shifts through time, the “abruptness” index was calculated through the time-series of each replicate microcosm in each experimental treatment (Fujita et al., 2023b). Specifically, an estimate of the abruptness index for time point *t* was obtained as the Bray–Curtis β-diversity between average community compositions from time points *t* − 4 to *t* and those from *t* + 1 to *t* + 5 (i.e., dissimilarity between 5-day time-windows). The Bray–Curtis β-diversity (Legendre and de Cáceres, 2013) was calculated as

$$\frac{\sum_{i=1}^{n}\left|X_{i\,j}-X_{i\,k}\right|}{\sum_{i=1}^{n}\left(X_{i\,j}+X_{i\,k}\right)},$$

where *X*_*ij*_ and *X*_*ik*_ denoted relative abundance of microbial amplicon sequence variant (ASV) *i* in the compared time windows (*j*, from *t* − 4 to *t*; *k*, from *t* + 1 to *t* + 5). An abruptness score larger than 0.5 indicates that turnover of more than 50% of community compositions occurred between the time-windows (Fujita et al., 2023b). Based on the calculated magnitude of time-series changes in community compositions (Fujita et al., 2023b; Figure 1A), we focused on a water-inoculum/oatmeal-medium replicate community showing the most abrupt (rapid and substantial) changes in community compositions among the 48 microbiomes examined as described in a study on metabolic interactions between species (Fujita et al., 2023a; Supplementary Figure 1).

### Shotgun metagenomics

**FIGURE 2:**
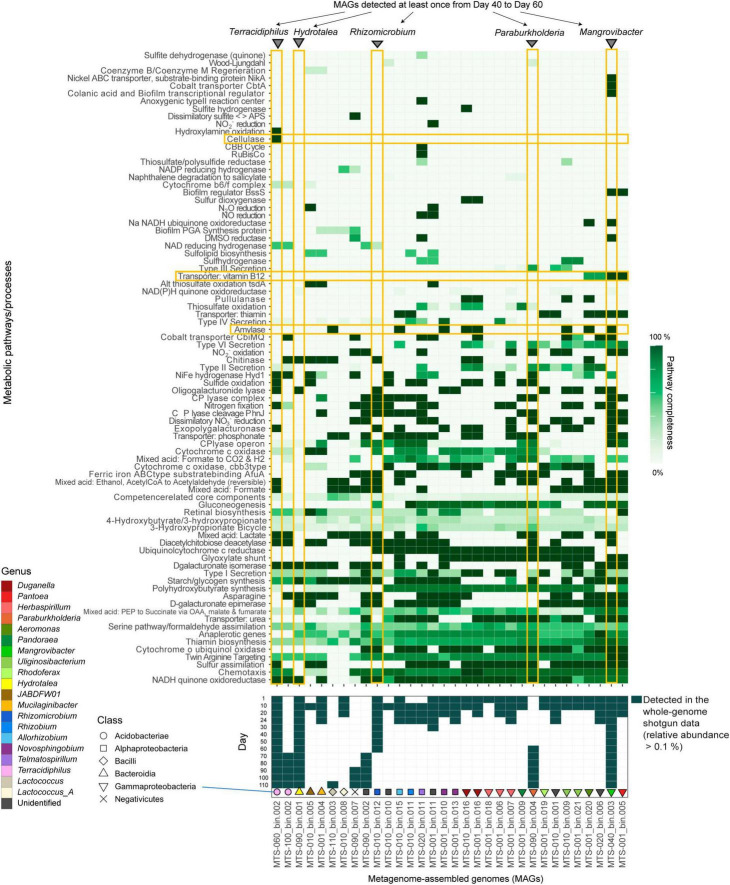
Metabolic pathway/process profiles of the MAGs. KEGG metabolic pathways/profiles of the reconstructed bacterial genomes (MAGs) are shown. The detection (relative abundance >0.1%) of each microbial MAG on each day within the shotgun metagenomic data is indicated in the panel below. Only the microbial MAGs with >80% completeness and <5% contamination were included (Supplementary Table 1). The five MAGs detected at least once from days 40–60 and metabolic pathways/processes mentioned in the main text (see section “Results”) are highlighted. Only the metabolic pathways/processes with highly heterogeneous patterns across microbial MAGs and those possessed by a small number of MAGs are shown. See Supplementary Figure 3 for detailed profiles of the metabolic pathways/processes.

Focusing on the replicate microcosm in which the most rapid and substantial turnover of community compositions was observed (replicate no. 5 of Water/Medium-A treatment; Figure 1A and Supplementary Figure 1), shotgun metagenomic sequencing was conducted by targeting 13 samples (day 1, 10, 20, 24, 30, 40, 50, 60, 70, 80, 90, 100, and 110) as described elsewhere (Fujita et al., 2023a). Specifically, each DNA sample was processed with Nextera XT DNA Library Preparation Kit (Illumina) and sequenced with the DNBSEQ-G400 (BGI; 200-bp paired-end sequencing). From the output data, sequencing adaptors were removed using Cutadapt (Martin, 2011) 2.5 and quality filtering was performed with Fastp (Chen et al., 2018) 0.21.0 (option settings: -q 20 -3 -W 6 -M 20; hereafter, default option settings were used unless remarked): ca. 10 Gb/sample was subjected to the analysis [in total, 159.96 Gb (1000.301 M reads)]. The sequences of each sample were assembled with metaSPAdes (Bankevich et al., 2012) 3.15.2 (option settings: -k 21,33,55,77,99,121). Binning was performed with metabat2, maxbin2, and concoct, and then bins with >50% completion and <5% contamination were reassembled using MetaWRAP (Uritskiy et al., 2018) 1.3.2, followed by quality assessing with CheckM (Parks et al., 2015) 1.1.3. The identity between metagenome-assembled genomes (MAGs) were calculated using FastANI (Jain et al., 2018) 1.33 and MAGs with >99% identity were dereplicated through the time-series (Supplementary Table 1). In the dereplication, the MAGs with the highest completeness and N50 statistics were selected as representative MAGs. Read-coverage was then calculated with CoverM (Woodcroft, 2021) 0.6.0, followed by taxonomic annotation was performed using GTDB-Tk (Chaumeil et al., 2020; Parks et al., 2022) 1.6. Only the MAGs with >80% completeness and <5% contamination were used in the downstream analyses [32 MAGs belonging to 20 genera (16 families; 12 orders)] (Fujita et al., 2023a; Figures 1B, C, 2, Supplementary Figure 2, and Supplementary Table 1). Gene annotation was performed with Prokka (Seemann, 2014) 1.14.6, yielding 6,999 annotated genes (Supplementary Data 1). To conduct additional functional annotation of genes, the orthology numbers of Kyoto Encyclopedia of Genomes (KEGG) were retrieved using GhostKOALA (Kanehisa et al., 2016) 2.2. For respective microbial MAGs (bins), completeness of metabolic pathways was estimated with KEGG decoder (Graham et al., 2018) 1.3. Based on the matrix representing KEGG metabolic pathway/process profiles of respective MAGs (Supplementary Data 2), a heatmap showing pathway/process completeness was drawn (Supplementary Figure 3).

### Background environmental conditions

For the 13 samples subjected to the shotgun metagenomic analysis, concentrations of ammonium (NH_4_^+^) and nitrate (NO_3_^–^) were measured to obtain Supplementary Information of background environmental conditions. Colorimetric methods with a modified indophenol reaction (Kandeler and Gerber, 1988; Hood-Nowotny et al., 2010) and the VCl3/Griess assay were applied for the measurements of NH_4_^+^ and NO_3_^–^, respectively. Samples were run in triplicates via a standard addition method to account for individual matrix effects (Taylor et al., 2007).

### Multivariate analysis of the metagenomic space

We used the shotgun metagenomic data to evaluate how the level of gene repertoire overlap among microbes shifted through time. We anticipated that microbial species with similar resource-use abilities or restrictions have similar genomic structure. Therefore, it is expected that species competing for the same resource tend to form clusters within the space defined based on the principal coordinate analysis (PCoA) of dissimilarity in gene repertoires. Thus, based on the whole matrix representing the presence/absence of the 6,999 genes annotated with the program Prokka (Seemann, 2014; Supplementary Data 1), the Jaccard metric of distance was calculated for each pair of the 32 microbial MAGs (*D*_*ij*_, where *i* and *j* represent MAGs). The Jaccard-distance estimates were then used to perform a PCoA. Using the obtained principal coordinate scores, all the microbial MAGs detected through the time-series were plotted on a multivariate space consisting of the first three PCoA axes (PCoA 1, PCoA 2, and PCoA 3). Since we did not have *a priori* knowledge of specific metabolic pathways keys to the microbe-to-microbe competition within the experimental microbiome, all datasets were included in this multivariate analysis. Given general characteristics of multivariate analysis based on β-diversity metrics, the multivariate reconstruction of ecological niche space depends greatly on the genes whose presence/absence profiles vary among species, while housekeeping genes possessed by most species are expected to contribute little to the multivariate analysis. To visualize the time-series shifts in the distribution of microbial genomes, the MAGs detected with the shotgun metagenomic sequencing (defined as the MAGs whose relative abundance is greater than 0.1%) at each time point was plotted on the three-dimensional space defined with the PCoA axes.

### Evaluation of niche overlap level

We quantitatively evaluated dynamics in the magnitude of community-scale niche overlap within the multivariate space. We developed two types of simple indices for evaluating community-scale niche overlap. The one is defined as the overall mean of gene-repertoire similarity between pairs of MAGs within a community. For a time point, the niche overlap index is calculated as:

$$\mathrm{niche}\,\mathrm{overlap}\,\mathrm{score}\,\left(\mathrm{overall}\,\mathrm{mean}\right)=1-\frac{\sum_{i\in T,j\in T,i\neq j}D_{i\,j}}{N_{T}\,\left(N_{T}-1\right)},$$

where *T* is the set of MAGs detected on a focal day (relative abundance >0.1%), *D*_*ij*_ is the Jaccard metric of dissimilarity (Anderson et al., 2011) in gene compositions, and *N_T_* is the number of MAGs detected on the day. By definition, this niche overlap value based on Jaccard dissimilarity varies from 0 (completely different repertoires of genes in all pairs of MAGs) and 1 (completely identical gene repertoires in all pairs of MAGs), allowing us to evaluate niche overlap levels of target communities within the standardized ranges. The other index is defined as mean value of gene-repertoire similarity with nearest neighbors. The alternative is calculated as:

$$\mathrm{niche}\,\mathrm{overlap}\,\mathrm{score}\,\left(\mathrm{nearest}\,\mathrm{mean}\right)=1-\frac{\sum_{i\in T,i\neq j}\min_{j\in T}\left(D_{i\,j}\right)}{N_{T}}.$$

This index can be modified by incorporating the information of the relative abundance of MAGs (*p_i_*) as follows:

niche⁢overlap⁢score⁢(weighted⁢nearest⁢mean)=

$$1-\sum_{i\in T,i\neq j}p_{i}\,\min_{j\in T}\left(D_{i\,j}\right).$$

To test whether a high level of fundamental-niche overlap is observed prior to drastic changes in microbial community structure, we examined relationship between the above niche overlap index and time-series shifts in community structure (Bray–Curtis β-diversity between present and next time points through the time-series of the shotgun metagenomic data).

## Results

### Functional dynamics of microbiomes

As indicated in the amplicon sequencing analysis (Fujita et al., 2023b; Figure 1A), drastic shifts from taxon-rich community states to oligopolistic states was observed around day 20 in the shotgun sequencing analysis (Figure 1B). After the drastic community compositional change, the system reached a quasi-stable state represented by the dominance of a *Hydrotalea* (Chitinophagaceae) bacterium (Figure 1B). The MAG of the *Hydrotalea* was characterized by relatively low GC content (38%) and relatively small genome size within the community (ca. 3.1 Mb; Figure 2). In contrast, the two bacterial MAGs consistently coexisted with the dominant *Hydrotalea* through the time-series (i.e., *Terracidiphilus* and *Mangrovibacter*) had larger genome size (4.2 and 5.4 Mb, respectively; Figure 1C), characterized by various genes absent from the *Hydrotalea* genome (Figure 2 and Supplementary Figure 3). Specifically, the *Terracidiphilus* MAG showed metabolic pathways/processes for degrading plant-derived biopolymers (e.g., cellulose; Figure 2). Meanwhile, the *Mangrovibacter* MAG had pathways/processes related to starch degradation (e.g., amylase) and vitamin-B_12_ transportation, which were absent from the genomes of *Hydrotalea, Terracidiphilus*, and the other MAG (*Rhizomicrobium*) detected on day 40–60 (Figure 2).

### Multivariate analysis of gene repertoires

**FIGURE 3:**
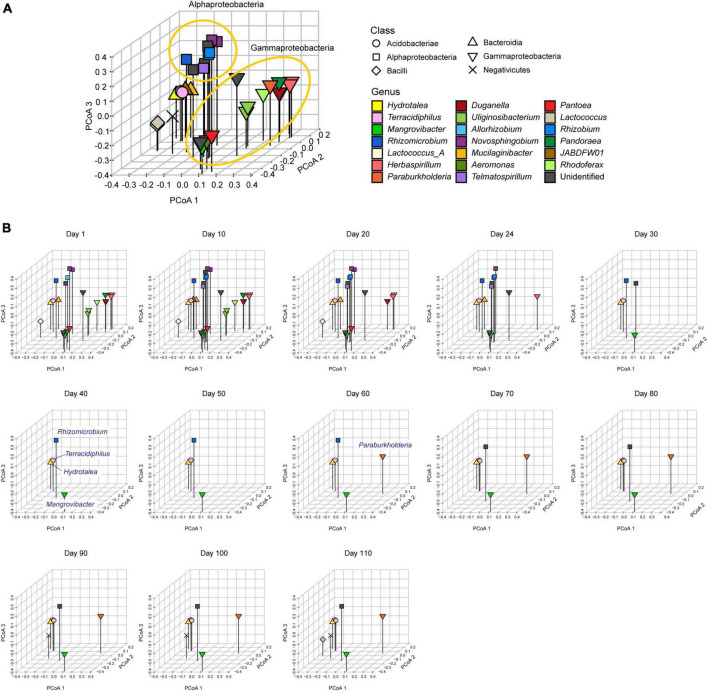
Metagenomic niche space. **(A)** Distributions of MAGs within metagenomic niche space. Based on dissimilarity in gene repertoires, microbial MAGs that appeared in the time-series of the target microcosm were plotted on the three-dimensional space defined by the principal coordinate analysis (PCoA) of 6,999 genes. **(B)** Changes in the distributions of microbial MAGs within niche space. At each time point, detected MAGs (relative abundance >0.1%) were plotted on the space defined in the multivariate analysis in the in the panel **(A)**.

Within the three-dimensional space defined with the gene repertoires (Figure 3A), alphaproteobacterial and gammaproteobacterial MAGs respectively constituted some clusters within the niche space reconstructed based on the multivariate analysis early in the microbiome dynamics (days 1–20; Figure 3B). This state with high niche overlap and potential within-guild competition for resources then collapsed into a simpler community state represented by *Hydrotalea*, *Mangrovibacter*, *Terracidiphilus*, and *Rhizomicrobium* as detailed above (Figure 3B). The space once occupied by many alphaproteobacterial and gammaproteobacterial MAGs remained unoccupied or sparsely occupied after the community compositional collapse. Even when the number of MAGs detectable with our shotgun-metagenomic sequencing increased again late in the time-series (four MAGs during days 40–50 vs. eight MAGs on day 110), dense aggregations of microbes with similar genomic compositions remained unobserved (Figure 3B).

### Metagenomic niche overlap

**FIGURE 4:**
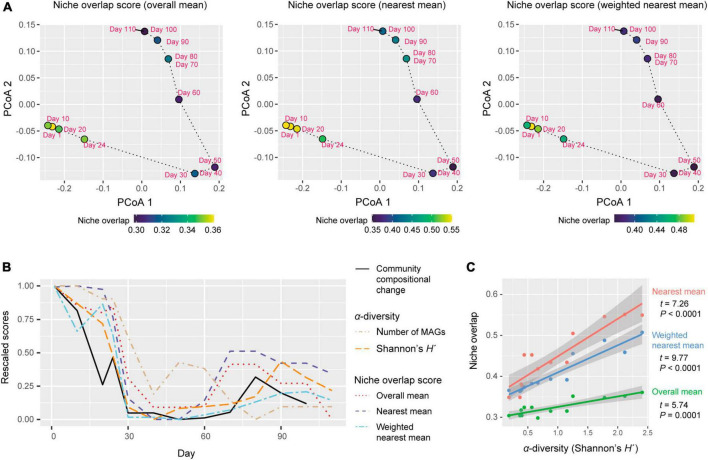
Dynamics of niche-overlap level. **(A)** Community-level profiles of metabolic pathways/processes and niche overlap index. The niche overlap indices were defined based on the Jaccard similarity/dissimilarity of gene compositions between pairs of the microbial MAGs detected at a target time point. Three types of niche overlap indices are shown on a PCoA surface representing community-level compositions of genes. On the PCoA surface, time points are distributed based on the sum of the gene repertoires of the detected MAGs. **(B)** Dynamics of niche-overlap levels. Niche overlap scores are shown across the time-series. The magnitude of community compositional changes (Bray–Curtis β-diversity between present and next time points through the time-series of the shotgun metagenomic data) and α-diversity indices of the communities are shown as well. The values of each index were rescaled between 0 (minimum through the time series) to 1 (maximum through the time series) through the time series. **(C)** Relationship between α-diversity and niche overlap scores. The lines represent linear regressions (with 95% confidence intervals).

**FIGURE 5:**
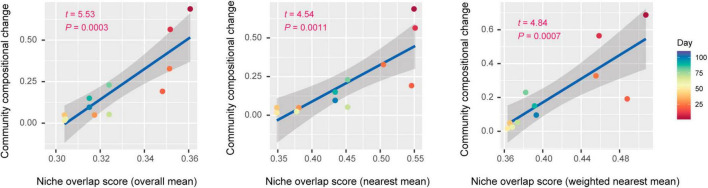
Niche overlap level and community compositional shifts. The magnitude of community compositional changes observed in the microbiome was regressed on each niche overlap index obtained based on the shotgun metagenomic analysis. Niche overlap index at each time point and time-series shifts in community structure (Bray–Curtis β-diversity between present and next time points through the time-series of the shotgun metagenomic data) are shown along horizontal and vertical axes, respectively. The regression lines are shown with 95% confidence intervals.

Each of three types of niche overlap indices was the highest on day 1 or day 10 and then it decreased until day 30 [overall mean, 0.361 (day 1); nearest mean, 0.551 (day 10); weighted nearest mean, 0.507 (day 1); Figure 4A]. Although the niche overlap score remained low between day 40 and 60 [overall mean, 0.304 (day 40–60); nearest mean, 0.349 (days 40 and 50) – 0.378 (day 60); weighted nearest mean, 0.363 (day 50) – 0.368 (day 60)], it increased again late in the microbiome time-series (Figures 4A, B). Note that α-diversity of the community showed similar temporal shifts and it was significantly associated with each of the three niche overlap indices (overall mean, *P* = 0.0001; nearest mean, *P* < 0.0001; weighted nearest mean, *P* < 0.0001; Figures 4B, C). Through the time-series, the estimated niche overlap level was significantly associated with the magnitude of the observed community compositional changes (overall mean, *P* = 0.0003; nearest mean, *P* = 0.0011; weighted nearest mean, *P* = 0.0007; Figure 5).

## Discussion

By developing simple metrics of among-species overlap of gene repertoires, we examined potential relationship between community-scale niche overlap and drastic changes in community structure. Early in the experimental microbiome dynamics, alphaproteobacterial and gammaproteobacterial species were present, resulting in relatively high niche-overlap scores at the community level (Figures 3, 4). The quasi-equilibrium state of microbial compositions then collapsed into another quasi-equilibrium represented by a small number of bacteria varying in genome size and metabolic capabilities. Throughout the time-series, higher niche overlap levels were associated with greater changes in microbial community compositions (Figure 5), although causation between the two variables deserves further experimental investigations. These findings lead to the working hypothesis that collapse of microbiome structure is predicted by the level of potential niche overlap within multivariate metagenomic space. In light of the “limiting similarity” rule of ecological niches (MacArthur and Levins, 1967), microbial species that exceed a critical limit of genome compositional similarity are expected to compete for the same resources intensively, eventually driving competitive exclusion processes. Thus, as examined in this study, the information of similarity/dissimilarity in genetically determined resource-use properties (i.e., fundamental niches) provides baselines for inferring consequences of interspecific interactions (Herold et al., 2020).

The results also indicated that niche overlap level does not necessarily show monotonic decrease through microbial community processes. Although gene-repertoire overlap level and detectable species richness sharply declined early in the microbiome dynamics, both variables gradually increased again around day 80 (Figures 1A, 4B). In the resurgence process, however, the dense clusters of alphaproteobacterial or gammaproteobacterial species detected until day 20 did not appear again within the niche space (Figure 3B). These observations suggest that once collapsed, microbial communities may not return to previous states with highest levels of niche overlap, but refilling of poorly used niches can occur under the constraint of limiting similarity within niche space. Although these insights are useful, our present analysis is based only on 13 time points of a microbiome experiment. Due to the limitation, it remained elusive to separate effects of α-diversity from those of gene-repertoire overlap (Figures 4B, C). Thus, the statistical analysis proposed in this study need to be expanded by reducing the cost of metagenomic sequencing as well as by developing more efficient pipelines for the computationally intensive analyses of metagenomic datasets.

The approach of systematically evaluating potential overlap of ecological niches have been previously explored in “community phylogenetics,” in which phylogenetic overdispersion/clustering is evaluated based on null model analysis of random assembly from species pools (Webb et al., 2002; Cavender-Bares et al., 2009; Mayfield and Levine, 2010). In those studies based on phylogenetic analyses, similarity of niches has been inferred based on the assumption that phylogenetically similar species have similar ecological properties (e.g., resource requirements). Nonetheless, given that convergent evolution of ecologically important traits is ubiquitous in the history of life (McCutcheon et al., 2009; Merhej et al., 2009; Marvig et al., 2015), the assumption of phylogenetic niche conservatism is not always met (Losos, 2008). Therefore, because gene repertoires are more direct proxies of species traits than phylogeny, metagenome-based analyses will deepen our understanding of community processes driven by competitive exclusion. Meanwhile, in the present analyses of gene repertoire overlap, we included whole metagenomic datasets of the examined microbes due to the lack of *a priori* insights into the metabolic pathways/processes playing essential roles in interspecific competition for resources. In this respect, our analysis is a preliminary conceptual step for evaluating potential overlap of fundamental niches at the community level. In future studies, analyses excluding housekeeping genes (Gibson et al., 2010; Maiden et al., 2013) or those focusing on specific functional groups of genes [e.g., carbohydrate degrading genes (Flint et al., 2012)] may provide more reliable inference of niche overlap (Herold et al., 2020). Moreover, antagonistic interactions between species will be more accurately evaluated by focusing on genes responsible for direct interspecific interactions, such as antibiotics production and antimicrobial resistance genes (Boolchandani et al., 2019). Because such selection of genes can critically influence threshold niche-overlap values for anticipating abrupt community compositional changes, setting a commonly applicable criterion of choosing target gene sets will help us perform comparative analyses across a wide range of microbial communities.

The simple framework for evaluating overlap of fundamental niches is applicable to diverse types of microbiomes. Given that our Jaccard-dissimilarity-based indices are standardized within the range from 0 to 1, the next crucial step is to examine how threshold niche overlap values for anticipating microbial community collapse vary among different types of ecosystems. Such threshold values can vary among ecosystems depending on their basic levels of sustainable functional redundancy. In our laboratory microbiome, for example, the lack of spatial structure (e.g., refuges for inferior species) and environmental fluctuations (e.g., temperature fluctuations) might have severely limited coexistence of functionally similar species (species with similar metabolic capabilities). In contrast, in human gut microbiomes, spatial complexity (Earle et al., 2015; Tropini et al., 2017) and temporally fluctuating environmental conditions (David et al., 2014) may reduce the risk of competitive exclusion, allowing higher levels of niche overlap within communities. Thus, extension of time-series metagenomic analyses to diverse types of ecosystems (Venter et al., 2004; Fierer, 2017; Jansson and Hofmockel, 2020; Trivedi et al., 2020) will enhance our knowledge of relationship among ecosystem properties, functional redundancy, and community stability. The accumulation of such knowledge will advance not only basic ecology but also applied microbiology. Insights into the dynamics of microbial species compositions and functional profiles, for example, will allow efficient selective enrichment of uncultivated microbes (Overmann et al., 2017) for industrial application.

While genomic information provides an ultimate platform for inferring fundamental niches (Palomo et al., 2018; Smith et al., 2019; Régimbeau et al., 2022), overlap of gene repertoires may not always result in competitive exclusion of species within communities. Even in a pair of species with similar gene repertoires, differentiation in gene expression patterns may occur to avoid overlap of resource-use patterns between species, allowing coexistence of the two species in an environment. Such differentiation of “realized niches (Chase and Leibold, 2004)” through phenotypic plasticity is potentially evaluated by transcriptomic or metabolomic analyses (Pereira and Berry, 2017; Muller, 2019; Nowinski and Moran, 2021; Malard and Guisan, 2023). Consequently, integration of (meta)transcriptome and (meta)metabolome analyses (Turner et al., 2013; Heintz-Buschart and Wilmes, 2018; Schirmer et al., 2018) with metagenome-based analyses (Herold et al., 2020) will reorganize our understanding of deterministic processes in microbiome dynamics.

## Data availability statement

The 16S rRNA sequencing data reported in a previous study (Fujita et al., 2023b) are available from the DNA Data Bank of Japan (DDBJ) with the accession number DRA013352, DRA013353, DRA013354, DRA013355, DRA013356, DRA013368, and DRA013379. The shotgun metagenomic data reported previous (Fujita et al., 2023a) are available with the DDBJ accession number DRA013382. The microbial community data are deposited at our GitHub repository (https://github.com/hiroakif93/MTS_nicheSpace). The matrices of the shotgun metagenomic data are available as Supplementary Data 1, 2. All the scripts used to analyze the data are available at the GitHub repository (https://github.com/hiroakif93/MTS_nicheSpace).

## Author contributions

HT: Conceptualization, Data curation, Funding acquisition, Investigation, Methodology, Project administration, Supervision, Validation, Visualization, Writing — original draft, Writing — review and editing. HF: Conceptualization, Data curation, Formal analysis, Funding acquisition, Investigation, Methodology, Project administration, Resources, Software, Validation, Visualization, Writing — original draft, Writing — review and editing. MU: Methodology, Writing — review and editing. KS: Methodology, Software, Writing — review and editing. MA: Methodology, Writing — review and editing. MY: Writing — review and editing. YO: Methodology, Writing — review and editing. AC: Investigation, Methodology, Writing — review and editing. IH: Writing — review and editing. KF: Methodology, Writing — review and editing. SF: Writing — review and editing. EK: Writing — review and editing.
