# Supplementary material for: Metagenomic analysis of ecological niche overlap and community collapse in microbiome dynamics
Source: Front Microbiol. 2023 Nov 15;14:1261137. doi: 10.3389/fmicb.2023.1261137 (PMC10684785; doi:10.3389/fmicb.2023.1261137)
Supplement: Supplementary file 1 [file Data_Sheet_1.PDF]

Supplementary Information for

Metagenomic analysis of ecological niche overlap and community collapse in microbiome dynamics

Hiroaki Fujita†, Masayuki Ushio, Kenta Suzuki, Masato S. Abe, Masato Yamamichi, Yusuke Okazaki, Alberto Canarini, Ibuki Hayashi, Keitaro Fukushima, Shinji Fukuda, E. Toby Kiers, and Hirokazu Toju†

†**Correspondence:** Hiroaki Fujita (fujita.h@ecology.kyoto-u.ac.jp) or Hirokazu Toju (toju.hirokazu.4c@kyoto-u.ac.jp).

This PDF file includes:

Supplementary Figures S1-3

Supplementary Table S1

Supplementary Information included in a separate file:

Supplementary Data S1-2

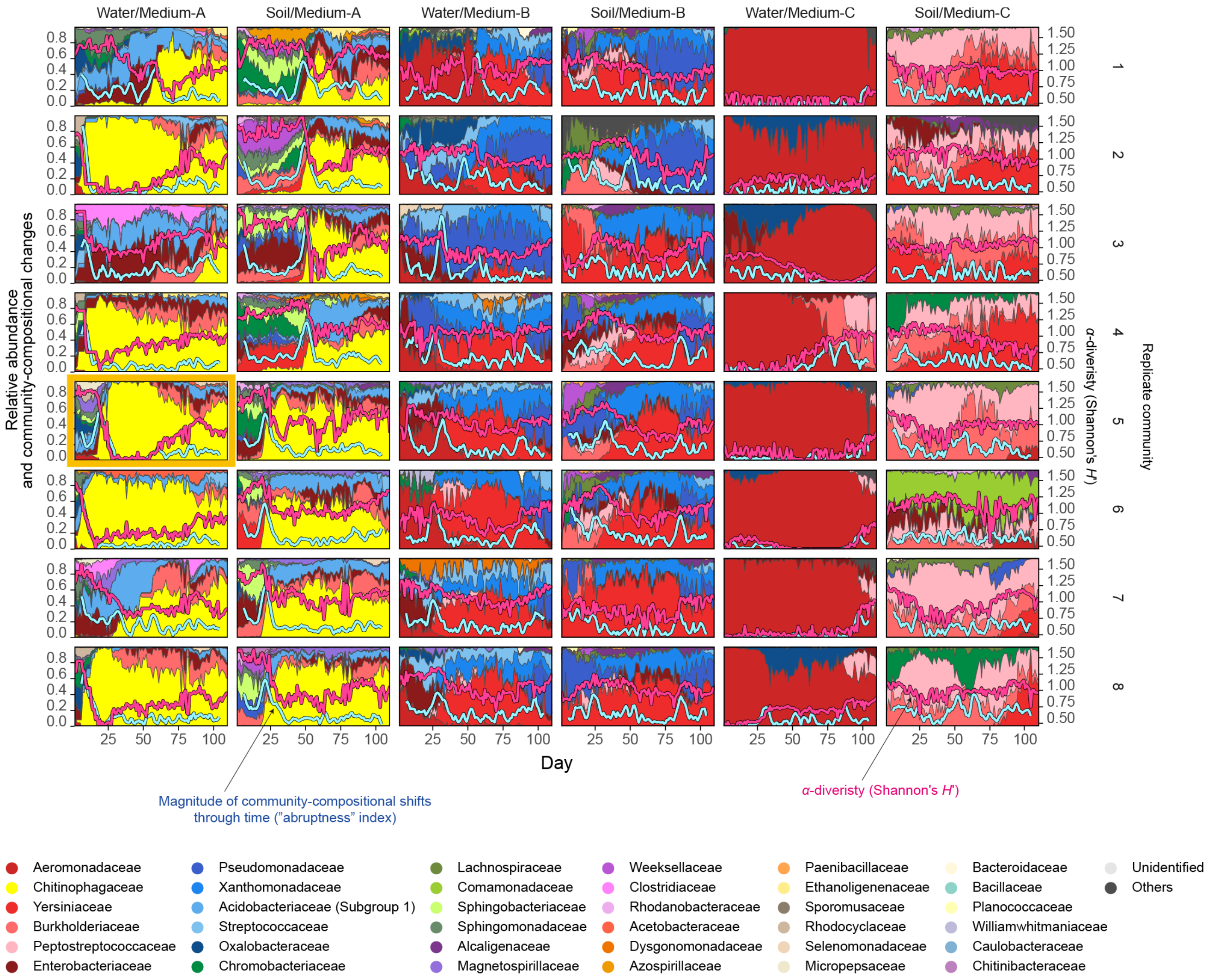

Supplementary Figure S1 | Dynamics of family-level community structure. The dynamics of microbial family-level compositions were visualized based on the 16S rRNA sequencing data of the previous study (Fujita et al., 2023b). The replicate microcosm (replicate no. 5 in Water/Medium-A treatment), which is subjected to the shotgun metagenomic sequencing analysis, is highlighted. The blue line represents the speed and magnitude of community compositional changes around each time point ("abruptness" index (Fujita et al., 2023b); see Methods). The red line indicates α-diversity (Shannon's H') of microbial ASVs (Fujita et al., 2023b). Reproduced from the data of a previous study (Fujita et al., 2023b).

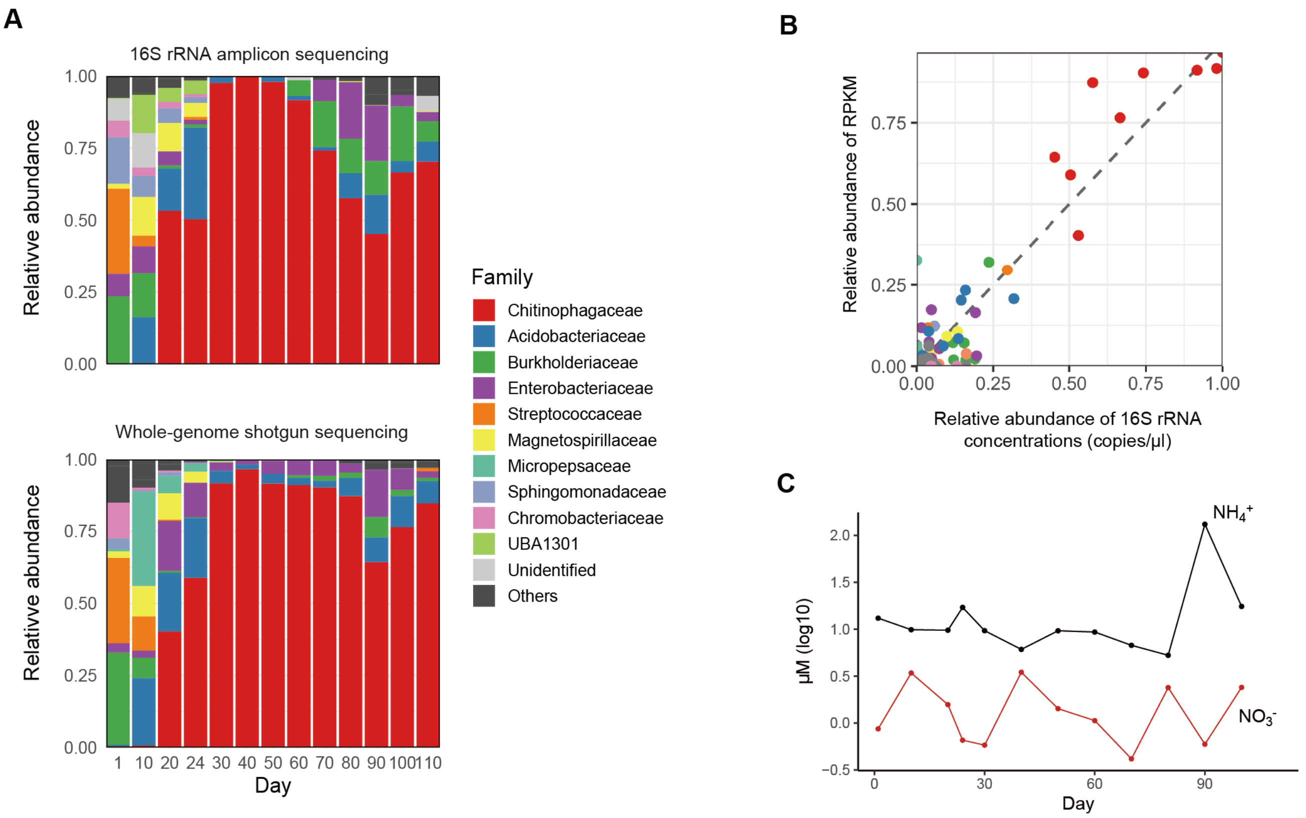

Supplementary Figure S2 | Overview of the shotgun metagenomic sequencing data. A Comparison of relative abundance of bacterial taxa (families) between 16S rRNA amplicon sequencing (Fujita et al., 2023b) (reproduced from the previous study (Fujita et al., 2023b); top) and shotgun metagenoimc sequencing (reproduced from the previous study (Fujita et al., 2023a); bottom). **B** Correlation between the family-level relative abundance of 16S rRNA and shotgun metagenomic sequencing data (Spearman's correlation; $\rho = 0.667$, df = 794, $P < 0.05$). Each point represents each family at each time point. **C** Background chemical properties. Changes in NO_3^- and NH_4^+ concentrations in the ecosystem are shown for the time points with shotgun metagenomic metagenomic data.

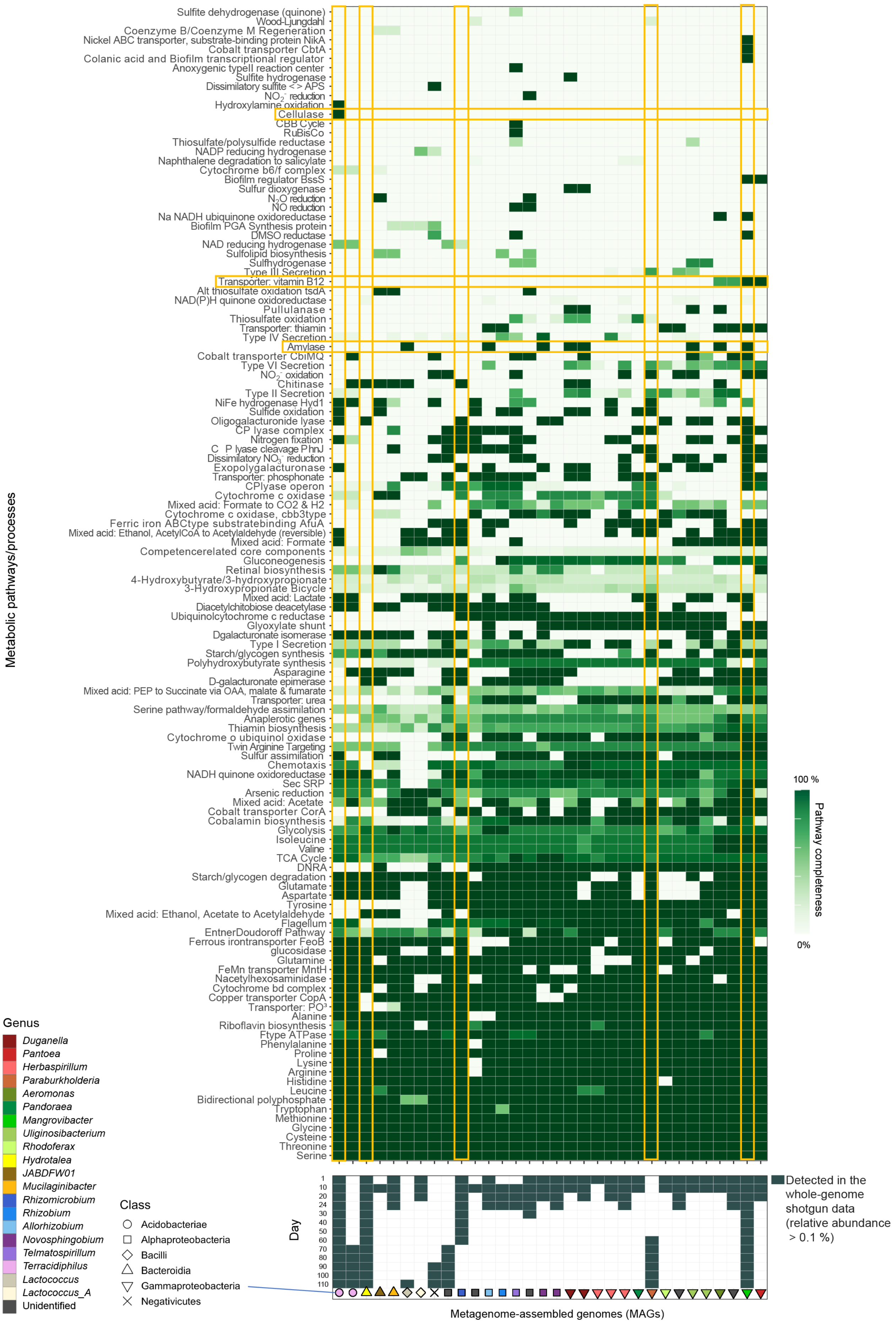

Sulfite dehydrogenase (quinone)
Wood-Ljungdahl
Coenzyme B/Coenzyme M Regeneration
Nickel ABC transporter, substrate-binding protein NikA
Cobalt transporter CbtA
Colanic acid and Biofilm transcriptional regulator
Anoxygenic typeII reaction center
Sulfite hydrogenase
Dissimilatory sulfite < > APS
NO2- reduction
Hydroxylamine oxidation
Cellulase
CBB Cycle
RuBisCo
Thiosulfate/polysulfide reductase
NADP reducing hydrogenase
Naphthalene degradation to salicylate
Cytochrome b6/f complex
Biofilm regulator BssS
Sulfur dioxygenase
N2O reduction
NO reduction
Na NADH ubiquinone oxidoreductase
Biofilm PGA Synthesis protein
DMSO reductase
NAD reducing hydrogenase
Sulfolipid biosynthesis
Sulfhydrogenase
Type III Secretion
Transporter: vitamin B12
Alt thiosulfate oxidation tsdA
NAD(P)H quinone oxidoreductase
Pullulanase
Thiosulfate oxidation
Transporter: thiamin
Type IV Secretion
Amylase
Cobalt transporter CbiMQ
Type VI Secretion
NO2- oxidation
Chitinase
Type II Secretion
NiFe hydrogenase Hyd1
Sulfide oxidation
Oligogalacturonide lyase
CP lyase complex
Nitrogen fixation
C P lyase cleavage PhnJ
Dissimilatory NO3- reduction
Exopolygalacturonase
Transporter: phosphonate
CPlyase operon
Cytochrome c oxidase
Mixed acid: Formate to CO2 & H2
Cytochrome c oxidase, cbb3type
Ferric iron ABCtype substratebinding AfuA
Mixed acid: Ethanol, AcetylCoA to Acetylaldehyde (reversible)
Mixed acid: Formate
Competencerelated core components
Gluconeogenesis
Retinal biosynthesis
4-Hydroxybutyrate/3-hydroxypropionate
3-Hydroxypropionate Bicycle
Mixed acid: Lactate
Diacetylchitobiose deacetylase
Ubiquinolcytochrome c reductase
Glyoxylate shunt
Dgalacturonate isomerase
Type I Secretion
Starch/glycogen synthesis
Polyhydroxybutyrate synthesis
Asparagine
D-galacturonate epimerase
Mixed acid: PEP to Succinate via OAA, malate & fumarate
Transporter: urea
Serine pathway/formaldehyde assimilation
Anaplerotic genes
Thiamin biosynthesis
Cytochrome o ubiquinol oxidase
Twin Arginine Targeting
Sulfur assimilation
Chemotaxis
NADH quinone oxidoreductase
Sec SRP
Arsenic reduction
Mixed acid: Acetate
Cobalt transporter CorA
Cobalamin biosynthesis
Glycolysis
Isoleucine
Valine
TCA Cycle
DNRA
Starch/glycogen degradation
Glutamate
Aspartate
Tyrosine
Mixed acid: Ethanol, Acetate to Acetylaldehyde
Flagellum
EntnerDoudoroff Pathway
Ferrous irontransporter FeoB
glucosidase
Glutamine
FeMn transporter MntH
Nacetylhexosaminidase
Cytochrome bd complex
Copper transporter CopA
Transporter: PO3
Alanine
Riboflavin biosynthesis
Ftype ATPase
Phenylalanine
Proline
Lysine
Arginine
Histidine
Leucine
Bidirectional polyphosphate
Tryptophan
Methionine
Glycine
Cysteine
Threonine
Serine
Metabolic pathways/processes
100 %
0%
Pathway completeness
Day
1
10
20
24
30
40
50
60
70
80
90
100
110
Detected in the whole-genome shotgun data (relative abundance > 0.1 %)
Metagenome-assembled genomes (MAGs)
Genus
Duganella
Pantoea
Herbaspirillum
Paraburkholderia
Aeromonas
Pandoraea
Mangrovibacter
Uliginosibacterium
Rhodoferax
Hydrotalea
JABDFW01
Mucilaginibacter
Rhizomicrobium
Rhizobium
Allorhizobium
Novosphingobium
Telmatospirillum
Terracidiphilus
Lactococcus
Lactococcus_A
Unidentified
Class
Acidobacteriae
Alphaproteobacteria
Bacilli
Bacteroidia
Gammaproteobacteria
Negativicutes

Supplementary Figure S3 | Detailed information of the metabolic pathway/process profiles of the MAGs. The KEGG metabolic pathways/processes of the reconstructed bacterial genomes (MAGs) are shown. The detection (relative abundance > 0.1 %) of each microbial MAG on each day within the shotgun metagenomic data is indicated in the panel below. Only the microbial MAGs with > 80 % completeness and < 5 % contamination were included (Supplementary Table 1). The five MAGs detected at least once from Day 40 to 60 and metabolic pathways/profiles mentioned in the main text are highlighted. See Figure 2 for the IDs of the MAGs. The detailed definition of the KEGG metabolic pathways/processes is available at https://github.com/bjtully/BioData/blob/master/KEGGDecoder/KOALA_definitions.txt.

Supplementary Table 1 | List of microbial MAGs detected in the whole-genome shotgun metagenomics. For each representative MAGs, estimates of completeness and contamination rates of the genome reconstruction as well as estimated genome size, GC nucleotide contents, coding density, and number of predicted genes are shown.

MAG ID	Phylum	Class	Order	Family	Genus	Species	Completeness (%)	Contamination (%)	Genome size (bp)	GC content (%)	Coding density (%)	Number of predicted genes
MTS-010_bin.016	Proteobacteria	Gammaproteobacteria	Burkholderiales	Burkholderiaceae	*Duganella*		99	0.33	5548277	62.5	91.6	4993
MTS-001_bin.010	Proteobacteria	Alphaproteobacteria	Sphingomonadales	Sphingomonadaceae	*Novosphingobium*		99.09	3.29	5636625	64.3	90.31	4976
MTS-001_bin.011	Proteobacteria	Alphaproteobacteria	Rhodospirillales	Magnetospirillaceae			99.38	0	3951389	62.6	91.44	3661
MTS-010_bin.001	Proteobacteria	Gammaproteobacteria	Burkholderiales	Chromobacteriaceae			99.57	0.85	4573844	52.1	85.31	4255
MTS-001_bin.013	Proteobacteria	Alphaproteobacteria	Sphingomonadales	Sphingomonadaceae	*Novosphingobium*		98.86	0.69	4198163	62.4	89.89	3913
MTS-010_bin.008	Firmicutes	Bacilli	Lactobacillales	Streptococcaceae	*Lactococcus_A*	*Lactococcus_A raffinolactis*	98.11	1.54	2372475	39.6	87.86	2327
MTS-040_bin.003	Proteobacteria	Gammaproteobacteria	Enterobacterales	Enterobacteriaceae	*Mangrovibacter*		99.56	0.27	5404296	52.2	87.61	5182
MTS-001_bin.016	Proteobacteria	Gammaproteobacteria	Burkholderiales	Burkholderiaceae	*Duganella*	*Duganella* sp003217375	99.92	1.19	6479266	62.6	92.81	5830
MTS-090_bin.001	Bacteroidota	Bacteroidia	Chitinophagales	Chitinophagaceae	*Hydrotalea*		98.52	0	3112189	38	85.06	2521
MTS-001_bin.018	Proteobacteria	Gammaproteobacteria	Burkholderiales	Burkholderiaceae	*Herbaspirillum*		84.16	0.74	3632523	54.3	89.11	3567
MTS-001_bin.019	Proteobacteria	Gammaproteobacteria	Burkholderiales	Burkholderiaceae	*Rhodoferax*		96.25	1.12	3077095	57	93.48	3084
MTS-010_bin.009	Proteobacteria	Gammaproteobacteria	Burkholderiales	Rhodocyclaceae	*Uliginosibacterium*		99.38	0.95	5092993	57	89.37	4744
MTS-001_bin.020	Proteobacteria	Gammaproteobacteria	Enterobacterales	Aeromonadaceae	*Aeromonas*	*Aeromonas hydrophila*	98.88	0.29	5006854	61.1	87.34	4561
MTS-001_bin.021	Proteobacteria	Gammaproteobacteria	Burkholderiales	Rhodocyclaceae	*Uliginosibacterium*		87.71	3.73	4570696	58.4	87.36	4787

MTS-020_bin.006	Proteobacteria	Gammaproteobacteria	Enterobacterales	Aeromonadaceae			100	0	3907279	49.1	90.11	3725
MTS-001_bin.004	Bacteroidota	Bacteroidia	Sphingobacteriales	Sphingobacteriaceae	*Mucilaginibacter*	*Mucilaginibacter oryzae*	97.62	0.4	5891481	44.3	87.99	5074
MTS-001_bin.005	Proteobacteria	Gammaproteobacteria	Enterobacterales	Enterobacteriaceae	*Pantoea*	*Pantoea* sp011752625	100	1.55	6025599	52.8	88.07	5560
MTS-001_bin.006	Proteobacteria	Gammaproteobacteria	Burkholderiales	Burkholderiaceae	*Herbaspirillum*		99.82	1.29	6377155	56.4	88.72	5610
MTS-001_bin.007	Proteobacteria	Gammaproteobacteria	Burkholderiales	Burkholderiaceae	*Herbaspirillum*		99.95	0.6	5673370	62.1	88.02	5070
MTS-010_bin.015	Proteobacteria	Alphaproteobacteria	Rhizobiales	Rhizobiaceae	*Allorhizobium*		98.31	0.84	5665159	55.1	88.93	5363
MTS-001_bin.009	Proteobacteria	Gammaproteobacteria	Burkholderiales	Burkholderiaceae	*Pandoraea*		99.55	0.96	6402802	63.5	88.08	5703
MTS-010_bin.010	Proteobacteria	Alphaproteobacteria	Rhizobiales	Phreatobacteraceae			82.3	0.98	3278745	59.7	90.8	3602
MTS-010_bin.011	Proteobacteria	Alphaproteobacteria	Rhizobiales	Rhizobiaceae	*Rhizobium*		96.14	1.99	6292370	60.5	87.24	6284
MTS-010_bin.012	Proteobacteria	Alphaproteobacteria	Micropepsales	Micropepsaceae	*Rhizomicrobium*		98.9	0.74	3592561	58.6	88.84	3388
MTS-020_bin.011	Proteobacteria	Alphaproteobacteria	Rhodospirillales	Magnetospirillaceae	*Telmatospirillum*		99.5	1.49	5604519	61.3	89.37	5178
MTS-010_bin.005	Bacteroidota	Bacteroidia	Chitinophagales	Chitinophagaceae	JABDFW01		88.53	0.25	4844654	44	87.52	4047
MTS-060_bin.002	Acidobacteriota	Acidobacteriae	Acidobacteriales	Acidobacteriaceae	*Terracidiphilus*		97.41	0.86	4157144	60.1	87.38	3415
MTS-090_bin.004	Proteobacteria	Gammaproteobacteria	Burkholderiales	Burkholderiaceae	*Paraburkholderia*	*Paraburkholderia tropica*	99.6	2.1	8236275	65	86.47	7367
MTS-100_bin.002	Acidobacteriota	Acidobacteriae	Acidobacteriales	Acidobacteriaceae	*Terracidiphilus*		99.14	0.86	4174131	58.4	87.74	3312
MTS-090_bin.002	Proteobacteria	Alphaproteobacteria	Acetobacterales	Acetobacteraceae			99.5	0.5	4089046	68.2	88.35	3679
MTS-090_bin.007	Firmicutes_C	Negativicutes	Selenomonadales	Selenomonadaceae			99.91	0.63	3554726	41.6	88.47	3344
MTS-110_bin.003	Firmicutes	Bacilli	Lactobacillales	Streptococcaceae	*Lactococcus*	*Lactococcus lactis*	99.62	0.51	2257933	34.9	87.27	2228

References

Fujita, H., Ushio, M., Suzuki, K., Abe, M. S., Yamamichi, M., Okazaki, Y., et al. (2023a). Facilitative interaction networks in experimental microbial community dynamics. *Front Microbiol* 14, 1153952. Available at: https://www.frontiersin.org/articles/10.3389/fmicb.2023.1153952.

Fujita, H., Ushio, M., Suzuki, K., Abe, M., Yamamichi, M., Iwayama, K., et al. (2023b). Alternative stable states, nonlinear behavior, and predictability of microbiome dynamics. *Microbiome* 11, 63.
